# Protein Palmitoylation and Its Role in Bacterial and Viral Infections

**DOI:** 10.3389/fimmu.2017.02003

**Published:** 2018-01-19

**Authors:** Justyna Sobocińska, Paula Roszczenko-Jasińska, Anna Ciesielska, Katarzyna Kwiatkowska

**Affiliations:** ^1^Laboratory of Molecular Membrane Biology, Department of Cell Biology, Nencki Institute of Experimental Biology of the Polish Academy of Sciences, Warsaw, Poland

**Keywords:** acyl-biotin exchange, bacterial effector proteins, click chemistry, fatty acylation of proteins, hemagglutinin, IFITM, *S*-palmitoylation, tumor necrosis factor α

## Abstract

*S*-palmitoylation is a reversible, enzymatic posttranslational modification of proteins in which palmitoyl chain is attached to a cysteine residue *via* a thioester linkage. *S*-palmitoylation determines the functioning of proteins by affecting their association with membranes, compartmentalization in membrane domains, trafficking, and stability. In this review, we focus on *S*-palmitoylation of proteins, which are crucial for the interactions of pathogenic bacteria and viruses with the host. We discuss the role of palmitoylated proteins in the invasion of host cells by bacteria and viruses, and those involved in the host responses to the infection. We highlight recent data on protein *S*-palmitoylation in pathogens and their hosts obtained owing to the development of methods based on click chemistry and acyl-biotin exchange allowing proteomic analysis of protein lipidation. The role of the palmitoyl moiety present in bacterial lipopolysaccharide and lipoproteins, contributing to infectivity and affecting recognition of bacteria by innate immune receptors, is also discussed.

## Introduction

Palmitic acid (C16:0) is a long-chain saturated fatty acid, and a component of various lipids playing important roles in cell membrane organization, signal transduction, and energy storage. Moreover, the palmitoyl chain can be attached to proteins in a process called palmitoylation (*S*-palmitoylation), which modification affects their localization and functioning.

In the human body palmitic acid is synthetized in a process called *de novo* lipogenesis. It takes place mainly in adipocytes, hepatocytes, and cells of lactating mammary glands (1). Palmitic acid is used for the synthesis of phospholipids and sphingolipids, may undergo elongation and/or desaturation into other fatty acids (e.g., stearic acid or oleic acid, respectively), and can be esterified to form storage lipids—triacylglycerols. Apart from *de novo* synthesis, palmitic acid is also provided to the human body with food. Since palmitic acid is universally found in natural fats, its consumption exceeds the consumption of other saturated fatty acids and, in the USA it accounts for about 60% of the total intake of saturated fatty acids (2). A growing body of experimental and clinical evidence points to a link between a westernized diet, including a high intake of saturated fatty acids, and chronic inflammatory diseases (3–5). As dietary saturated and unsaturated fatty acids apparently modulate activity of immune cells, their influence on the immune responses triggered upon infection is also beginning to be investigated (6). These facts drive the interest in palmitic acid with an aim of elucidating the molecular mechanisms of its immunomodulatory properties.

In this review, we focus on *S*-palmitoylation of proteins crucial for the interactions of pathogenic bacteria and viruses with the host. We emphasize novel data on the role of *S*-palmitoylated proteins in the invasion of host cells by pathogens and those involved in the host innate immune responses to the infection, which have been obtained thanks to the application of new technical approaches. Recently, substantial progress in the understanding of protein palmitoylation was made possible by the development of methods allowing high-throughput analysis of cellular/tissue palmitoyl proteomes. We begin, however, by showing how unique protein *S*-palmitoylation is among other protein lipidations.

## The Many Faces of Fatty Acylation of Proteins

### *S*-Palmitoylation of Proteins and Its Influence on Protein Localization, Trafficking, and Stability

**Figure 1:**
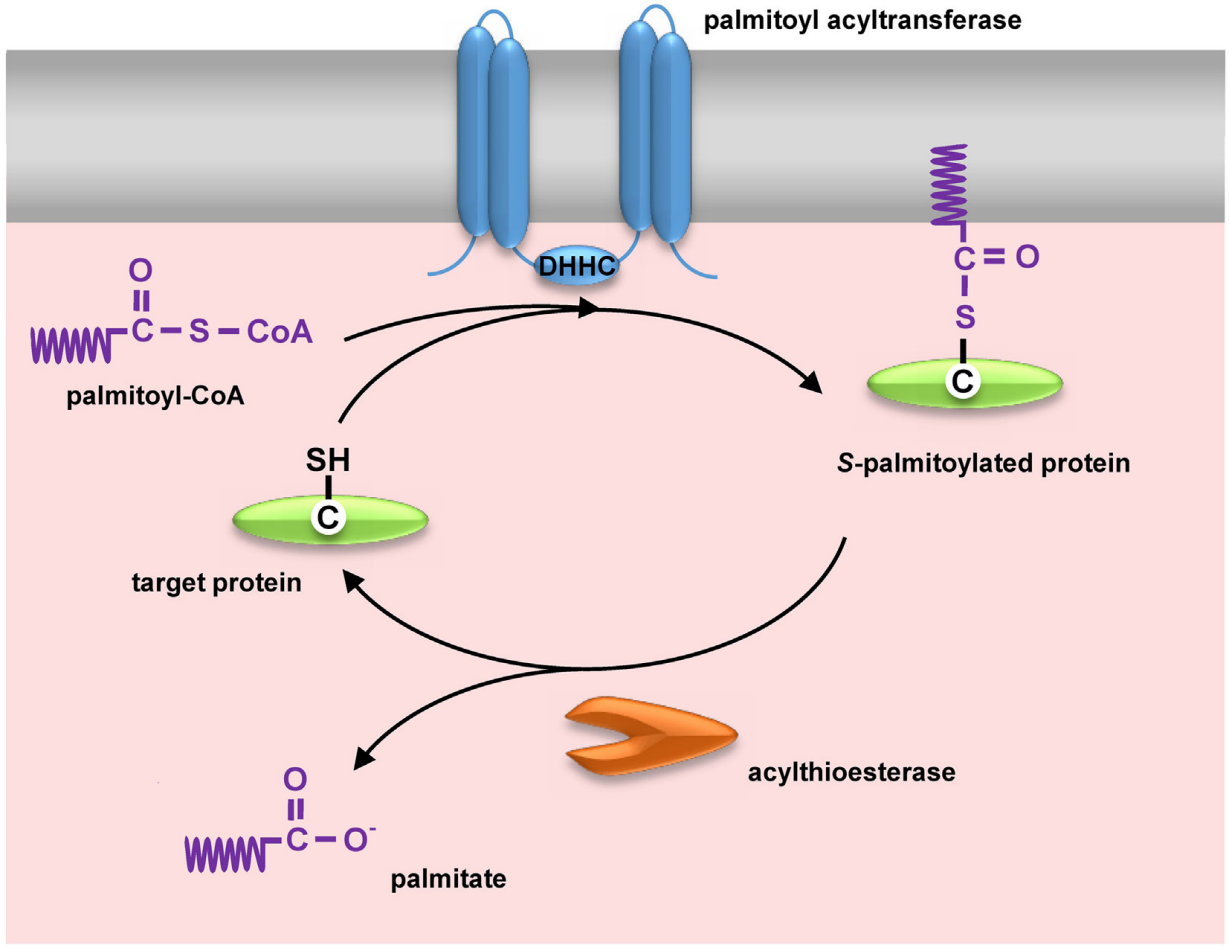
Dynamic protein *S*-palmitoylation. Palmitate is transferred to the thiol group of cysteine from palmitoyl-CoA by integral membrane zDHHC-family palmitoyl acyltransferases. Upon *S*-palmitoylation cytosolic proteins gain a hydrophobic moiety allowing their anchoring in the membrane. Proteins are depalmitoylated by acylthioesterases [acyl-protein thioesterase (APT) 1/APT2 and ABHD17] and translocate to the cytosol.

*S*-palmitoylation is a posttranslational modification of proteins consisting in a potentially reversible covalent attachment of palmitoyl chain to a cysteine residue(s) of proteins through a thioester bond (Table 1). Thus, *S*-palmitoylation resembles other reversible regulatory posttranslational protein modifications, including phosphorylation or acetylation, well-established factors affecting protein structure and functions. In particular, *S*-palmitoylation modifies cellular localization of proteins and their stability. The most dramatic changes of localization concern cytosolic proteins which upon *S*-palmitoylation acquire a hydrophobic anchor facilitating their docking into membranes (Figure 1). However, several integral membrane proteins also undergo *S*-palmitoylation. It often occurs on cysteine residue(s) located in the proximity of the junction of the transmembrane and cytoplasmic domains of the protein. *S*-palmitoylated transmembrane proteins occupy various cellular compartments, such as endoplasmic reticulum, Golgi apparatus, and the plasma membrane. In accordance, for some proteins, such as transmembrane adaptor proteins in leukocytes, *S*-palmitoylation was found secondary to the length and hydrophobicity of the transmembrane domain as a determinant of plasma membrane destination (7).

**Table 1:** Fatty acylation and prenylation of proteins.

Modification	Lipid	Amino acid modified	Linkage	Representative proteins	Reference
*S*-acylation	C16:0 Palmitic acid	Cysteine 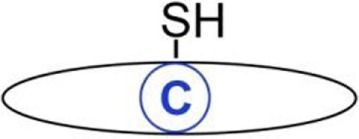	Thioester	IFITM3, toll-like receptor 2, hemagglutinin (HA), glycoprotein G of vesicular stomatitis virus, Lyn, and other Src kinases	(8–12)
	C18:0 Stearic acid			HA and transferrin receptor	(9, 13)
	C16:1 Palmitoleic acid			IFITM3	(14)
	C18:1 Oleic acid			H-Ras	(14, 15)
	C20:4 Arachidonic acid			Fyn kinase	(15)

*N*-acylation	C14:0 Myristic acid^a^	Glycine 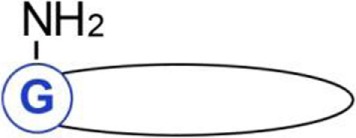	Amide	Gag of human immunodeficiency virus-1, Lck, and other Src kinases, Arf1	(16–19)
	C16:0 Palmitic acid			Gαs	(20)

	C16:0 Palmitic acid	Cysteine 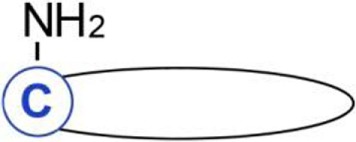	Amide	Sonic hedgehog^b^	(21)

ε-*N*-acylation	C14:0 Myristic acid	Lysine 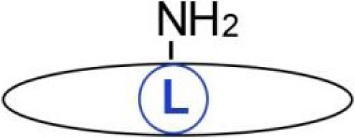	Amide	Tumor necrosis factor α, interleukin-1, and α-hemolysin of *Escherichia coli*	(22, 23)
	C16:0 Palmitic acid			Adenylate cyclase of *Bordetella pertussis*	(24)

*O*-acylation	C16:0 Palmitic acid	Serine or threonine 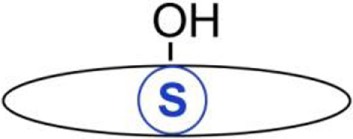	Oxyester	Histone H4	(25)
	C8:0 Octanoic acid			Ghrelin	(26)
	C16:1 Palmitoleic acid			Wnt proteins, e.g., Wnt3a^c^	(27)

*S*-prenylation	Farnesyl	Cysteine 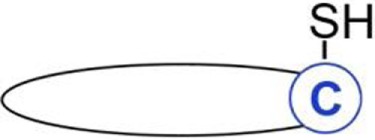	Thioether	H- and N-Ras	(28)
	Geranylgeranyl			Rab proteins	(28)

*Attachment of glycosylphosphatidylinositol anchor or phosphatidylethanolamine (29) to the C-terminus of proteins is also a form of lipidation but is not shown here*.

*^a^*N*-myristoylation is in most cases co-translational, but during apoptosis caspases can cleave some proteins, such as BID, exposing their N-terminal glycine residue, which is then modified by attachment of myristate (30)*.

*^b^Hedgehog proteins are additionally modified by covalent attachment of cholesterol to their C-terminus (31)*.

*^c^*O*-acylation of Wnt proteins is reversed by Notum of the α/β hydrolase superfamily (31)*.

*S*-palmitoylation also contributes to the compartmentalization of proteins to distinct domains of membranes—rafts and tetraspanin-rich microdomains. In fact, the interest in *S*-palmitoylation was boosted when it was found to be required for the targeting of some signaling proteins to rafts. Rafts are nanodomains of the plasma membrane and some intracellular membranes, mainly of the *trans*-Golgi apparatus, rich in sphingolipids, glycerophospholipids with saturated fatty acid chains, and cholesterol (32). The plasma membrane nanodomains are sites of signal transduction by distinct receptors of immune cells involved in both acquired immune reactions, such as T cell receptor (TCR), Fcε receptor I, Fcγ receptor II, and in innate immune responses, such as toll-like receptor 4 (TLR4) (33, 34). Rafts are also sites of virion assembly and budding, as established, e.g., for influenza A virus and human immunodeficiency virus-1 (HIV-1) (35, 36). Peripheral membrane proteins acylated with saturated fatty acids are likely to anchor preferentially between the ordered saturated lipids of rafts rather than between the disordered lipids of the surrounding membrane. It has been shown that, owing to their raft localization, *S*-palmitoylated kinases of the Src family interact with raft-associating plasma membrane immunoreceptors and initiate signaling cascades fundamental to acquired immunity (15, 37, 38). It is worth noting that also the acyl chains attached to proteins can affect the membrane structure. Studies on model membranes have revealed that palmitic and myristic acids facilitate formation of ordered lamellar membrane regions (39, 40). In accordance, *S*-palmitoylation of erythrocyte peripheral membrane protein called membrane-palmitoylated protein 1 (MPP1) was found to be required for the proper lateral organization and fluidity of erythrocyte membrane. In the absence of MPP1 *S*-palmitoylation, raft assembly was disturbed and erythrocyte functioning compromised leading to hemolytic anemia in patients deficient in the enzyme catalyzing this reaction (41, 42). Preferential raft association is a feature of some *S*-palmitoylated transmembrane proteins, e.g., adaptor proteins PAG, LAT, and NTAL, which collaborate with the abovementioned immunoreceptors. In fact, palmitoylation is required for the raft association of most integral raft proteins (8, 43, 44).

On the other hand, *S*-palmitoylation does not obligatorily confer raft localization on transmembrane proteins. Certain *S*-palmitoylated proteins, such as transferrin receptor, glycoprotein G of vesicular stomatitis virus (VSV), and anthrax toxin receptor, tumor endothelial marker 8 (TEM8), are actually excluded from rafts. Apparently, a combination of *S*-palmitoylation and the properties of the transmembrane domain of the protein contribute to its destination to the raft or non-raft environment (43, 45). It has also been proposed that the attachment of a fatty acyl chain at the juxtamembrane cysteine(s) of a protein can induce tilting of its transmembrane fragment, determining in which part of the membrane it will accommodate to avoid a hydrophobic mismatch potentially caused by the thickness of the bilayer (46).

That not all *S*-palmitoylated proteins associate with rafts has been shown convincingly for macrophage-like RAW264 cells, where only about half of those proteins were found in the Triton X-100-resistant membrane fraction enriched in rafts (47, 48). In accordance, proteomic data on the distribution of *S*-palmitoylated proteins in prostate cancer cells have revealed that several such proteins are recovered in the non-raft (Triton X-100-soluble) fraction and are likely localized to microdomains enriched in scaffold proteins called tetraspanins (49). The tetraspanins are small integral membrane proteins found in the plasma membrane and other cellular membranes, having four transmembrane helices and undergoing *S*-palmitoylation at several conserved cysteine residues. The tetraspanins interact with each other and with various transmembrane and cytosolic partners, often also *S*-palmitoylated, forming microdomains (“tetraspanin web”) (50). It has been suggested that the amino acid composition of the *S*-palmitoylation site in some transmembrane proteins, such as the adaptor proteins involved in acquired immune responses, determines the association of those *S*-palmitoylated proteins with rafts or with the tetraspanin-enriched microdomains (44). An intriguing and still poorly addressed question concerns the relation between rafts and the tetraspanin-enriched microdomains, apparently of functional significance, e.g., during virus budding from host cells (35). This uncertainty stems partially from the fact that *S*-palmitoylation of tetraspanins governs their interactions with cholesterol and gangliosides leading at certain conditions to the recovery of tetraspanins in detergent-resistant membrane fractions enriched in rafts (51, 52). Besides its involvement in targeting proteins to rafts or tetraspanin-enriched microdomain, *S*-palmitoylation has been found to govern accumulation of the transmembrane chaperone protein calnexin in the perinuclear domain of endoplasmic reticulum (53).

*S*-palmitoylation also affects protein stability through its interplay with ubiquitination or phosphorylation, as found for the anthrax toxin receptor TEM8, antiviral interferon-induced transmembrane protein IFITM1, calnexin, and zDHHC6, one of palmitoyl acyltransferases described below (54–57).

Possibly the most intriguing is the reversible character of *S*-palmitoylation. Enzymes catalyzing palmitoylation and depalmitoylation of proteins have been characterized (58, 59). Palmitate is transferred onto the thiol group of cysteine from cytosolic palmitoyl-CoA by palmitoyl acyltransferases, enzymes containing the zinc finger DHHC domain named after the highly conserved Asp–His–His–Cys peptide (Figure 1). This is a two-step reaction comprising transient autoacylation of zDHHC enzymes and transfer of the fatty acyl chain from this intermediate to a protein substrate (60). In mammals, the zDHHC enzyme family consists of 24 proteins, and zDHHC proteins are also found in other eukaryotes but not in bacteria nor are they encoded by viral genomes. Mammalian zDHHC enzymes, each having at least four transmembrane helices, are located in the plasma membrane, endoplasmic reticulum, and Golgi apparatus (58). They display some specificity toward their protein substrates and also selectivity toward fatty acyl moieties other than palmitate, which contributes to the heterogeneity of lipids attached to proteins, such as viral glycoproteins described below (61). In the opposite process, the thioester bond is cleaved by acyl-protein thioesterases (APTs) (APT1 and APT2) and palmitoyl protein thioesterases (PPTs) (PPT1 and PPT2), which are localized in the cytosol and in lysosomes, respectively. APT1 and APT2 likely govern the dynamic functional changes of *S*-acylation of proteins (62) while PPT1 and PPT2 depalmitoylate proteins during their degradation (63, 64). Recently, serine hydrolases of the ABHD17 family have also been identified as depalmitoylating enzymes, and their specific substrate proteins determined (65, 66). Of note, the zDHHCs, APT1/APT2, and ABHD17 proteins are *S*-palmitoylated themselves, and palmitoylation of zDHHCs and depalmitoylation of APT1/2 can occur in a cascade manner (57, 62).

The dynamic cycles of palmitoylation/depalmitoylation detected for several peripheral membrane proteins are often synchronized with intracellular trafficking of those proteins. They circulate between the plasma membrane and the Golgi apparatus or endosomes, as exemplified by N- and H-Ras, R7-regulator of G protein and APTs. In fact, it is proposed that palmitoylation-dependent anchoring of APT1 in the plasma membrane allows it to depalmitoylate H-Ras at this location, while subsequent auto-depalmitoylation releases APT1 guiding it, alongside H-Ras, for another round of palmitoylation at the Golgi apparatus (62, 67–69). Cycles of palmitoylation/depalmitoylation are crucial for signaling by distinct plasma membrane receptors and for their distribution (69–71). Activation of TCR receptor or Fas receptor in T cells was found to trigger quick and transient palmitoylation of Lck kinase of the Src family (72, 73), but the exact meaning of the dynamic protein *S*-palmitoylation for processes triggered during the host–pathogen interaction awaits elucidation.

It is worth mentioning that although the zDDHC enzymes catalyze bulk protein palmitoylation in eukaryotic cells (74), some proteins have a unique autopalmitoylation activity. These include Bet3, a component of a multisubunit transport protein particle complex involved in vesicular trafficking, TEA domain transcription factors, and also bacterial Evf protein (75–78). The palmitic acid residue is attached constitutively to a specific cysteine residue of those proteins, remains buried inside a hydrophobic pocked in their core thereby affecting the tertiary structure and, thus, interactions with other proteins (75, 77). An exhaustive discussion on the physiology of *S*-palmitoylated proteins in eukaryotic cells can be found in several recent reviews (46, 79, 80).

### *S*-Palmitoylation Is a Special Case of *S*-Acylation of Proteins

It has been established that, in addition to palmitate, various other fatty acyl moieties, such as saturated stearate (C18:0) or monounsaturated palmitoleate (C16:1), and oleate (C18:1) can be attached *via* the thioester linkage to proteins. The early reports on the heterogeneity of the fatty acyl moieties attached to cysteines obtained by analysis of selected immunoprecipitated proteins (15, 16, 81–83) have recently been complemented by a comprehensive proteomic analysis of fatty-acylated proteins of macrophage-like RAW264 cells (14). The latter study showed that an enrichment of culture medium of cells with monounsaturated fatty acids leads to their incorporation into a similar set of proteins as those normally modified with palmitate. Among them, several proteins relevant to innate immune responses were found. All these data justify the use of a broader term *S*-acylation rather than *S*-palmitoylation (Table 1). The physiological consequences of *S*-acylation of proteins with individual fatty acids are slowly being revealed. Modification of Fyn kinase with polyunsaturated fatty acid residue, such as arachidonate (C20:4), disturbed its raft localization and, thereby, TCR signaling (15). A heterogeneity of *S*-acylation was also found in viral spike proteins, such as hemagglutinin (HA) of influenza A virus, and E1 and E2 of Semliki Forest virus, which are modified in host eukaryotic cells by attachment of both palmitate and stearate (9). In HA, stearate is attached at the transmembrane cysteine while palmitate is attached to two cysteine residues in a membrane-proximal region of the protein. The stearoyl chain seems to accommodate into a groove formed by amino acids of the transmembrane helix shaping the domain in a way that facilitates its fitting into rafts (84). *S*-stearoylation of human transferrin receptor 1 at the juxtamembrane cysteine residues(s) is a key factor of the signaling cascade controlling mitochondrial morphology and functioning (13). Of interest, the latter study also showed that dietary supplementation of stearic acid reversed the deleterious effects of a genetically determined mitochondria dysfunction in *Drosophila*. Taking into account that unsaturated fatty acids affect the profile of *S*-acylation of proteins *in vitro* (14, 15), it is of outmost interest whether a similar effect of unsaturated and saturated (palmitic) fatty acids could be achieved *in vivo* with respect to proteins of immune cells.

### *N*- and *O*-Acylation of Proteins

Beside *S*-acylation, less frequently palmitate can also be attached to the amine group of various amino acids (glycine, cysteine, and lysine) giving *N*-palmitoylation or to the hydroxyl group of serine or threonine in a process called *O*-palmitoylation (Table 1). As during *S*-palmitoylation, also other fatty acids can be utilized in these processes named then *N*- and *O*-acylation. Thus, a type of protein *N*-acylation is *N*-myristoylation, a frequent modification contributing to membrane anchoring of peripheral proteins. The saturated myristate (C14:0) is transferred to the protein from myristoyl-CoA by *N*-myristoyl transferase (two isozymes in mammals). In a vast majority of cases, myristate is attached co-translationally to the N-terminal glycine residue (after removal of the initiator methionine) *via* an amide linkage (Table 1). Like most lipidations, this modification is irreversible. Several viral proteins are *N*-myristoylated, such as Gag of HIV-1 crucial for budding of newly formed virions from plasma membrane rafts of host cells, and proteins of parasitic protozoa *Plasmodium falciparum, Trypanosoma brucei*, and *Leishmania donovani* (causing malaria, African sleeping sickness, and leishmaniosis, respectively). For this reason, *N*-myristoyl transferase is considered a potential drug target in the therapy of these diseases (17–19, 85, 86).

Data on the *N*- and *O*-palmitoylation of proteins involved in the host–pathogen interactions are limited, but interesting conclusions can be drawn from the information concerning proteins taking part in other processes. *N*-palmitoylation of the N-terminal glycine of the α-subunit of a heterotrimeric G protein (Gαs) has been described (20) (Table 1) besides the well-known *S*-palmitoylation of this pivotal signaling protein. The *N*-palmitoylation of Gαs is irreversible, and the enzyme responsible for this modification is unknown. It has been speculated that *S*- to *N*-palmitoyl migration can occur both *in vivo* and also *in vitro* during mass spectrometry analysis (20, 87). This suggests that caution is needed in interpreting results of this methodological approach, which is used with increasing frequency to study fatty acylation of proteins in immune cells (see next sections). Probably the best-characterized is the *N*-palmitoylation of the N-terminal cysteine residue of hedgehog proteins (sonic, Indian, and desert in mammals). It determines secretion of these proteins, which regulate embryonic patterning (Table 1). Secreted Wnt and ghrelin proteins are examples of *O*-acylation of serine residues with unusual fatty acid residues such as palmitoleate (C16:1) and octaonoate (C8:0) (Table 1). The fatty acylation of hedgehog, Wnt, and ghrelin is catalyzed by enzymes from the multipass membrane-bound *O*-acyl transferases family (31). Besides these unusual fatty acid residues, attachment of palmitate to serine and threonine residues is found in secreted venom toxins of the spider *Plectreurys tristis*, which selectively target neuronal ion channels (88). Also histone H4 is *O*-palmitoylated at a serine residue in the nucleus by acyl-CoA:lysophosphatidylcholine acyltransferase (25) (Table 1). The latter is of special interest in the context of innate immune responses since histone H4 *O*-palmitoylation regulates transcriptional activity, which is the final outcome of the pro-inflammatory signaling pathways triggered by receptors of the innate immune system.

Special attention should be devoted to ε-*N*-acylation consisting in the attachment of a fatty acid residue to the side chain of lysine by amide linkage (Table 1). ε-*N*-myristoylated are interleukin 1α (IL-1α) and tumor necrosis factor α (TNFα), the pro-inflammatory cytokines crucial in combating bacterial infections (22). The enzyme(s) catalyzing this reaction is unknown, but it has been established that sirtuins reverse this modification (89). The ε-*N*-acylation affects the release of TNFα by immune cells (90, 91). Surprisingly, this rare modification is also found in toxins of so-called RTX (repeats-in-toxin) class released by some pathogenic Gram-negative bacteria (23, 24). We describe these cases in more detail in the following sections.

### *S*-Prenylation, Another Common Lipidation of Proteins

Besides *S*-palmitoylation and *N*-myristoylation, *S*-prenylation is another common lipidation that endows proteins with a hydrophobic moiety and contributes to their association with membranes. This modification relies on the posttranslational and irreversible attachment of either farnesyl or geranylgeranyl chains to a cysteine residue in the C-terminal CAAX box (alternatively also CC and CXC motifs) *via* a thioether linkage. The process is catalyzed by protein prenyl transferases that use polyprenylpyrophosphate as the donor of the isoprenoid group (Table 1). In peripheral membrane proteins, the *S*-palmitoylation site is often located in proximity of *N*-myristoylation or *S*-prenylation sites or a polybasic motif, which all are likely to mediate initial weak binding of a protein to a membrane and thereby facilitate subsequent attachment of palmitate to the protein by the integral membrane zDHHC enzymes (31). In contrast to *S*-palmitoylation, data on the role of *S*-prenylation of proteins key to the host–pathogen interactions are scarce (92). However, since *S*-prenylation is typical for the ubiquitous small GTPases of Ras superfamily, it is vital for proper functioning of B and T cells (93, 94).

A glance at Table 1 indicates that palmitate can be covalently bound *via* oxyester, amide, and thioester linkages to respective amino acid residues creating an array of possible modifications. *O*- and *N*-palmitoylation of proteins seems to be stable, resembling in this regard the other common protein lipidations, *N*-myristoylation and *S*-prenylation. By contrast, there exist enzymes cleaving the thioester bond formed during *S*-palmitoylation. For a long time, our understanding of protein *S*-palmitoylation and its dynamics was poor in comparison with other reversible protein modifications due to technical difficulties. Only recently have these difficulties been overcome with the introduction of methods allowing high-throughput identification of palmitoylated proteins, also those involved in the immune response to microbial pathogens, as discussed in the next sections.

## Methodological Progress Facilitates Detection of Protein Palmitoylation

One of the basic problems hindering studies on protein *S*-palmitoylation lies in the fact that there is no identifiable consensus sequence for the palmitoylation site that could facilitate its prediction. From the technical point of view, the progress in a comprehensive survey of protein *S*-palmitoylation was also hampered by a lack of antibodies detecting this modification, with the sole exception of an antibody specific to palmitoylated PSD-95 (95). A classical method used to demonstrate protein palmitoylation is based on metabolic labeling of living cells with [^3^H]-palmitic acid, subsequent immunoprecipitation of a selected protein and detection of the incorporated tritiated fatty acid by autoradiography (96). A major disadvantage of this method is its low sensitivity. Only a minute fraction of the radioactive palmitate is bound to proteins, the majority being incorporated into lipids, which requires lengthy film exposure (counting in days).

A methodological breakthrough in the identification of palmitoylated proteins came with the development of two non-radioactive methods based on so-called click chemistry (97–99) and acyl-biotin exchange (ABE) (74, 100). These techniques have paved the way for high-throughput mass spectrometry-based proteomic analysis of protein palmitoylation in various cells and tissues and facilitated identification of new palmitoylated proteins of both pathogens and host cells involved in the innate immune responses.

### The Click Chemistry-Based Method of Analysis of Protein Fatty Acylation

**Figure 2:**
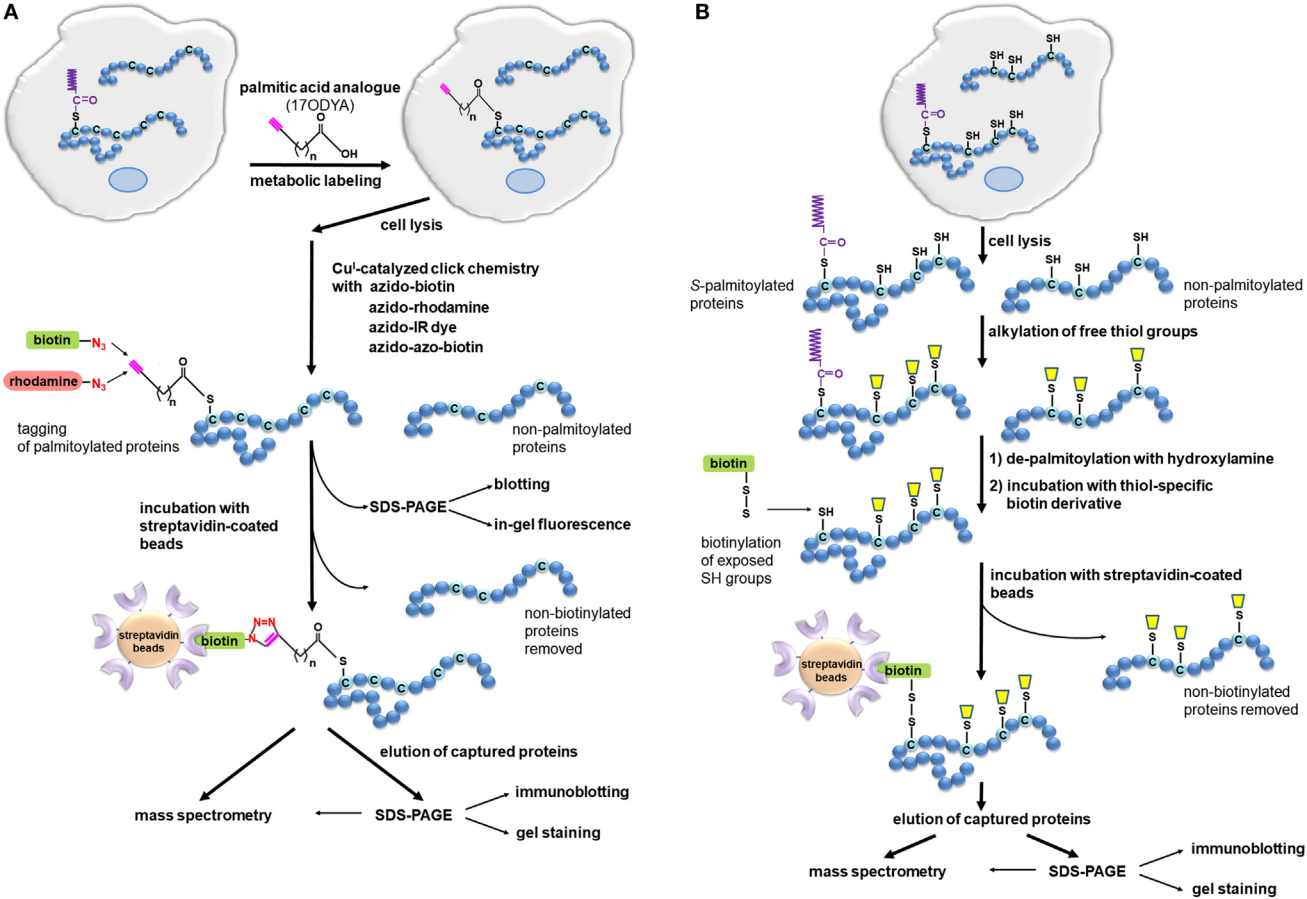
Detection of *S*-palmitoylated proteins using click chemistry and acyl-biotin exchange (ABE). **(A)** Click chemistry-based method. Cells are metabolically labeled with an alkyne-functionalized palmitic acid analog, such as 17-octadecynoic acid (17ODYA), and after cell lysis, the click reaction is conducted with azido-tagged biotin or fluorescent probes allowing enrichment and detection of labeled proteins in various ways. Biotinylated proteins can be bound on a streptavidin resin and then released using, e.g., high concentrations of urea and SDS (108). When a cleavable derivative of biotin, azido-azo-biotin, is used the labeled proteins are eluted from streptavidin beads with sodium dithionite, which cleaves the diazobenzene moiety in the linker arm of azido-azo-biotin, and analyzed by mass spectrometry or immunoblotting (109). **(B)** ABE method. Cells or tissues are lysed, free thiol groups of proteins are blocked by alkylation, and palmitoyl moieties are released with hydroxylamine. The newly exposed protein thiol groups are subjected to labeling with biotin-HPDP allowing selective binding, elution, and analysis of the originally *S*-palmitoylated proteins. The proteins can also be captured without biotinylation through a direct interaction of their thiol residues with a thiol-reactive resin (acyl-RAC technique).

In the click chemistry-based method, cells are metabolically labeled with a palmitic acid analog bearing an alkyne group at the ω carbon of the fatty acyl chain, such as 17-octadecynoic acid (17ODYA) or alk-16 (Figure 2A), and this step resembles the classic labeling of cells with [^3^H]-palmitic acid. However, in the click chemistry-based assay, the labeling and lysis of cells is followed by *in vitro* coupling of the function group of the palmitic acid analog to a reporter tag, which greatly enhances the sensitivity of detection of labeled proteins (98, 99). Thus, after cell lysis, the labeled proteins are subjected to Cu^(I)^-catalyzed cycloaddition known as “click” reaction with an azide-bearing detection tag. In this step, a triazol is formed between the alkyne group in the palmitic acid analog and the azide of the tag (Figure 2A). The azide-bearing tags can be either fluorescent, such as tetramethylrhodamine or dyes with infrared fluorescence, or carry a biotin moiety. Depending on the tag used, subsequent SDS-PAGE separation of proteins allows global visualization of palmitoylated proteins by simple in-gel fluorescence or by blotting with a streptavidin-conjugated reporter (98, 101, 102). Notably, proteins biotinylated *via* the click reaction can also be enriched on streptavidin-coated beads and then subjected to on-bead tryptic digestion (or in-gel digestion if eluted from the beads) followed by identification by mass spectrometry. Such comprehensive click chemistry-based proteomic analysis has brought about identification of an array of palmitoylated proteins in dendritic cells (10, 103), macrophage-like RAW264 cells (14), and T cells (99, 104, 105). Some of the *S*-palmitoylated proteins newly identified in those studies, such as IFITM3 and TLR2, are involved in the host–pathogen interactions regulating innate immune responses (10, 103), while many others are known to contribute to adaptive immunity (99, 105), as described below. Recently, global profiling of *Toxoplasma gondii* (the causative agent of toxoplasmosis) has been performed revealing that many components of the parasite’s motility complex are palmitoylated (106). Similar studies on *Cryptococcus neoformans* (the fungus causing cryptococcal meningitis) have revealed a contribution of specific zDHHC palmitoyl acyltransferase, called Pfa4, to its virulence (107). Moreover, application of analogs of various saturated and unsaturated fatty acids confirmed the heterogeneous nature of the fatty acylation of proteins in RAW264 cells and suggested that dietary unsaturated fatty acids, after incorporation to proteins, can change their properties and thereby affect the functioning of immune cells (14).

The major advantage of the click chemistry-based method is that it can reveal the time course of protein *S*-palmitoylation. By using click chemistry-based labeling in the pulse-chase mode, one can follow the dynamics of protein palmitoylation. With such an approach, it was found that the palmitate turnover on Lck, an Src-family tyrosine kinase, is accelerated by T cell activation (72). Additional introduction of stable isotope labeling by amino acids in cells (SILAC) has provided quantitative proteomic data on the dynamics of protein palmitoylation in the cell (104, 110). This approach revealed, rather unexpectedly, that in unstimulated T cell hybridoma, the palmitoylation of most protein species does not undergo turnover (104). Another advantage of the click chemistry-based assay is its high specificity, because the alkyne group introduced in the analog of palmitic acid is not normally found in cells (98, 102). The click chemistry-based methods can also be used to follow the cellular localization of palmitoylated proteins by immunofluorescence when combined with the proximity ligation technique (111, 112). Palmitoylation of individual proteins can also be studied after their immunoprecipitation (11, 72, 73, 98).

Despite its unquestionable success, the click chemistry-based methods have limitations. They will detect only those proteins that undergo palmitoylation during the period of the metabolic labeling of cells. One should also bear in mind that the palmitic acid analog can be incorporated at *S*-, *N*-, and *O*-palmitoylation sites alike (111, 112). In addition, although 17ODYA (alk-16) is preferentially used to mimic palmitoylation of proteins, it can also be incorporated with low efficiency at *N*-myristoylation sites of proteins (98, 99). Another group of proteins that will be labeled with the palmitic acid analog but are not *S*-palmitoylated are those bearing the glycosylphosphatidylinositol (GPI) anchor (85, 113). Most of these limitations can be overcome using various fatty acid reporters, inhibitors, and by exploiting the sensitivity of the thioester bond to hydroxylamine treatment. Given the large variety of chemical reporters preferentially mimicking distinct fatty acids, recent years have witnessed a plethora of chemistry-based proteomic studies not only on palmitoylated but also myristoylated proteins and proteins bearing the GPI anchor, including those of pathogens and immune cells (10, 14, 85, 86, 114).

### The ABE Method Reveals Protein *S*-Acylation

The ABE method can be used as a complement to the click chemistry-based approach in cell studies but unlike the latter it is uniquely suitable for studying whole tissues. ABE does not require metabolic labeling of proteins in living cells, thus some of the abovementioned limitations and difficulties do not apply. The ABE method relies on *in vitro* exchange of thioester-linked palmitate to a derivative of biotin which allows subsequent affinity purification of the resulting biotin-labeled proteins on streptavidin-coated beads (Figure 2B). The first step of the ABE involves lysis of cells or tissues followed by irreversible blockage of free thiol groups in the solubilized proteins by alkylation, most often with *N*-ethylmaleimide. Subsequently, the thioester bonds existing in *S*-palmitoylated proteins are broken with hydroxylamine, releasing palmitoyl moieties. The newly exposed thiol groups can now be tagged with sulfhydryl-reactive derivatives, such as biotin-HPDP, forming disulfide bonds with thiols. The biotinylated proteins are subsequently captured on streptavidin-coated beads and eluted with agents that reduce the disulfide bond between the protein and biotin-HPDP, such as β-mercapthoethanol, DTT, or TCEP (49, 74, 115, 116). As an alternative to biotinylation, in the so-called acyl-RAC technique, the newly exposed protein thiol groups in hydroxylamine-treated cell lysates are captured on a resin containing sulfhydryl-reactive groups (117). In both ABE and acyl-RAC, the eluted proteins can be separated by SDS-PAGE and visualized by gel staining or immunoblotting, or identified by mass spectrometry. Furthermore, when the hydroxylamine-released palmitoyl moieties are exchanged for a polyethylene glycol-maleimide derivative of a distinct molecular weight, a shift in-gel migration of tagged proteins is observed reflecting the number of fatty acyl residues originally *S*-bound to the protein (118, 119).

The ABE method has so far been used successfully for proteomic profiling of *S*-acylated proteins in immune cells, such as RAW264 cells (48), several types of blood cells, such as platelets, primary T cells, and immortalized B cells (120–122), pathogenic microorganisms such as *T. brucei* and *T. gondii* (123, 124), and tissues (125, 126). To quantify the aberrations in protein palmitoylation in a mouse model of Huntington’s disease, whole animal stable isotope labeling of mammals (SILAM) was applied followed by tissue isolation and ABE procedure (127). In another approach, for quantitative analysis of the T-cell palmitoylome, ABE was combined with labeling of proteins with various oxygen isotopes during their digestion with trypsin before mass spectrometry analysis (122). In addition, preselection of tryptic peptides obtained by ABE on streptavidin-coated or sulfhydryl-reactive resins greatly facilitates the identification of *S*-acylation sites by mass spectrometry (49, 110, 117).

Some aspects of the ABE method deserve a comment. Since the assay relies on the sensitivity of thioester bonds to hydroxylamine, ABE detects all *S*-acylation without distinguishing between *S*-palmitoylation and the other cases. Furthermore, there is a possibility of false-positive detection of proteins bearing a thioester linkage with compounds other than fatty acyl residues, such as ubiquitin in the E2 ubiquitin conjugase Ubc1 (115). Another source of false-positives is proteins in which free thiol groups were not completely alkylated before biotinylation. On the other hand, insufficient deacylation of *bonafide* fatty-acylated proteins with hydroxylamine results in their absence in the final sample (116).

In summary, the click chemistry-based method relies on metabolic labeling of cells with a palmitic acid analog which incorporates into proteins and next tagging it with reporter molecules greatly enhancing the sensitivity of detection. It only reveals proteins undergoing *S*-palmitoylation during metabolic labeling of cells and allows revealing turnover of this modification. By contrast, the ABE method is based on direct binding of sulfhydryl-reactive derivatives to thiol groups of cysteines unraveled by hydroxylamine treatment after lysis of cells or tissues. It allows the investigation of the whole but static palmitoylome. A comparative proteomic study of protein palmitoylation in *P. falciparum* found that the sets of proteins identified using these two approaches overlapped in 57.2% (113), indicating that they provide complementary data on the cellular palmitoyl proteome. Thanks to the application of the click chemistry- and ABE-based methods numerous new palmitoylated proteins have been identified. In 2015, a SwissPalm database was launched, (128) which provides an excellent, manually curated resource of information on palmitoylated proteins, palmitoylation sites, etc., available at http://swisspalm.epfl.ch/. All these efforts have greatly furthered our knowledge on molecular mechanisms regulating diverse aspects of cell functioning, including host–pathogen interactions and progress of infectious diseases, as highlighted below.

## Palmitate as a Component of Proteins and Lipids Related to Bacterial Pathogenicity

Bacteria lack protein palmitoyl acyltransferases of the zDHHC family and, therefore, are essentially devoid of *S*-palmitoylated proteins. Yet, they have developed unique mechanisms utilizing fatty acids, such as palmitic acid, to modify their glycolipids and proteins. These modifications augment infectivity and help bacteria evade recognition by the host innate immune system. For example, the vast majority of Gram-negative bacteria produce lipopolysaccharide (LPS) as a part of their outer membrane. LPS is composed of the variable polysaccharide *O*-antigen and more-conserved lipid A containing two glucosamine residues hexa-acylated with hydroxymyristic, myristic, and lauric acid. Lipid A is recognized by CD14 protein and TLR4 receptor complexed with MD2 protein on the plasma membrane of the host immune and some non-immune cells. Activation of TLR4 triggers strong pro-inflammatory reactions aiming at eradication of the bacteria, but when exaggerated, eventually leading to sepsis (129). Incorporation of an additional palmitoyl chain into lipid A markedly diminishes its ability to activate TLR4 and to induce the host pro-inflammatory responses, which is correlated with an increased survival of bacteria forming a biofilm (130, 131). This strategy is utilized among others by *Salmonella typhimurium*, a causative agent of gastroenteritis, by *Bordetella bronchiseptica*, a respiratory pathogen of human and other mammals, and by *Yersinia pestis* causing plague (132, 133). The formation of the extra-acylated LPS relies on the transfer of palmitate from phospholipids onto the hydroxymyristate chain at position 2 of glucosamine of lipid A. The reaction is catalyzed by lipid A palmitoyltransferases (PagP in *Salmonella* and its homologs in other bacteria) localized in the outer membrane of these pathogens (134, 135). In addition to causing steric hindrance preventing the binding to the TLR4/MD2 complex, the hepta-acylation of LPS also protects bacteria from the lytic activity of cationic antimicrobial peptides, most likely by reducing the fluidity of the bacterial outer membrane (136, 137).

Apart from being incorporated into LPS in diverse bacteria, palmitate has also been found to modify a virulence factor of Gram-negative *Erwinia carotovora*, the Evf protein. The palmitoyl chain is linked *via* a thioester bond to the Cys209 residue at the center of Evf, plausibly by a self-palmitoylating activity of the protein. *E. carotovora* is a phytopatogen using insects such as *Drosophila* as vectors for dissemination between plants. The palmitoylation of Evf is required for infectivity of *E. carotovora* and its persistence in the insect gut, however, its mode of action of unknown. It has been speculated to be linked with an ability of Evf to associate with lipid bilayers, but the lack of similarities between Evf and any other bacterial protein of known function makes prediction on this subject difficult (79).

A number of bacterial toxins of so-called RTX class released during infection of mammals by pathogenic Gram-negative bacteria undergo ε-*N*-acylation of the side chain of internal lysines. These toxins include adenylate cyclase of *Bordetella pertussis*, acylated with palmitic acid, and α-hemolysin of extraintestinal (uropathogenic) *Escherichia coli*, acylated with myristic acid and also 15- and 17-carbon fatty acids. The acylation is catalyzed by an endogenous bacterial acyltransferase which, unlike its eukaryotic counterparts, transfers the acyl chain not from acyl-CoA but from acyl-carrier protein. The acylated toxins secreted by the bacteria bind to the plasma membrane of the host cells, oligomerize and form pores causing cell lysis. In the case of the toxin of *B. pertussis*, essential is also the delivery of the adenylate cyclase moiety to the cell interior. Acylation is required for virulence possibly being involved in oligomerization of the toxins (23, 24, 138).

Although lacking *S*-palmitoylated proteins (with the single known exception of Evf), bacteria express a wide range of membrane-bound proteins modified by a complex lipidation at the N-terminus, with palmitate frequently being a component of the lipid moiety (139, 140). The bacterial lipoproteins are synthesized in a multistep process catalyzed by a unique set of lipoprotein processing enzymes, Lgt, LspA, and Lnt, absent in eukaryotic cells. The formation of these lipoproteins begins with the attachment of a diacylglycerol *via* a thioester bond to a cysteine residue located in the so-called lipobox motif of the signal sequence of the transmembrane lipoprotein precursor. The signal sequence is then cleaved next to the lipid-modified cysteine leaving it at the N-terminus of the mature protein (141). In Gram-negative and less frequently also Gram-positive bacteria, a third fatty acid residue is additionally attached *via* an amide linkage to the amino group of the cysteine in a reaction analogous to the *N*-acylation of hedgehog proteins (see Table 1). This di- and tri-lipidation ensures membrane anchoring of the lipoproteins. All such lipoproteins of Gram-positive bacteria are exposed to the milieu while in Gram-negative bacteria some face the periplasm. The lipoproteins of Gram-positive bacteria, e.g., *Streptococcus pneumoniae* (causing pneumonia), *Mycobacterium tuberculosis* (tuberculosis), and Gram-negative bacteria, such as *Neisseria meningitidis* (meningitis), *Y. pestis* (plague), the spirochaete *Borrelia burgdorferi* (Lyme disease) and *Treponema pallidum* (syphilis) are crucial for their virulence. They control several aspects of the host–pathogen interactions, like adhesion and entry to host cells, protection against proteolysis and oxidative stress in the host cell, and regulation of expression of genes encoding cytokines both during initiation and progress of the disease (140–142). The surface exposure of the lipoproteins allows their involvement in the host cell invasion while on the other hand forming the so-called pattern signal recognized by the TLR2 receptor, which triggers the pro-inflammatory responses helping to combat the bacteria (143). Of interest, TLR2 is *S*-palmitoylated, as discussed below. The involvement of lipoproteins in pathogenesis fuels studies on their properties. One such recent work employing click chemistry to profile the lipoproteins of *E. coli* identified 88 lipoproteins with high/medium confidence, 70% of them predicted before by bioinformatics analysis (144). Notably, in that study a 14-carbon alkynyl fatty acid analog alk-14 rather than alk-16 was preferentially incorporated into the lipoproteins, contradicting earlier studies using gas chromatography and TLC, which found that palmitate was predominantly used for bacterial protein modification (139). Further studies are required to establish whether the fatty acid found in lipoproteins varies depending on culture conditions or is species specific. For example, 17ODYA labeling for click reaction confirmed incorporation of palmitate into pallilysin (Tp0751), a lipoprotein of *T. pallidum*. Pallilysin is a metalloprotease that degrades human fibrinogen and laminin. It is suggested that its exposure on the bacteria surface enables degradation of host structural proteins to facilitate rapid dissemination of this highly invasive pathogen (140).

Bacteria occasionally high-jack the palmitoylation machinery of host cells to modify the environment so as to favor their internalization, survival, and replication inside the cells. *Bacillus anthracis* (the causative agent of anthrax) is an example of such bacteria that modify *S*-palmitoylation of host proteins to their ends. The anthrax toxin produced by this pathogen binds to the TEM8 and CMG2 (capillary morphogenesis protein-2) proteins which, under physiological conditions, are involved in cell–cell and cell–extracellular matrix interactions. They are *S*-palmitoylated at multiple (two to four) cysteines (54). The *S*-palmitoylation of TEM8 was found to inhibit its association with plasma membrane rafts preventing its ubiquitination by the raft-associated E3 ubiquitin ligase Cbl. The binding of anthrax toxin drives association of the receptor-toxin complexes with rafts possibly correlated with depalmitoylation of the receptor. This allows subsequent ubiquitination of the receptor, an uptake of the receptor/toxin complexes in a clathrin-dependent manner and eventual delivery of the toxin to endosomes. These events are facilitated by *S*-palmitoylation of partner(s) of the receptors, most likely including kinases of the Src family (54, 145, 146).

While *B. anthracis* utilizes palmitoylated host proteins to induce its internalization, a growing body of data suggests that also bacterial proteins can undergo *S*-palmitoylation inside the host cells. This type of modification concerns so-called effectors, bacterial proteins that are injected into the host cell cytoplasm either across the plasma membrane or the membrane of vesicles enclosing internalized pathogens, with the help of their secretion systems. These are secretion systems type III and type IV, homologs of which have been described for pathogens and symbionts of mammals, insects, and plants (147, 148). The bacterial effectors can be *S*-palmitoylated to reach host cell membranes and thereby accumulate at a location most suitable for their activity. Application of the click chemistry-based method utilizing an analog of palmitic acid (alk-16) for cell labeling has revealed *S*-palmitoylation of two effector proteins of *Salmonella enterica*, such as SspH2 and SseI (149). *S. enterica* invades gut endothelial cells and is a leading cause of gastroenteritis and typhoid fever. SspH2 carries an E3 ubiquitin ligase domain while SseI shows sequence homology to bacterial proteins that have a deamidase activity, and inhibits migration of *Salmonella*-infected cells. The latter activity requires *S*-palmitoylation of SseI. Both proteins are stably *S*-palmitoylated, most likely by zDHH3 and zDHH7 of the host and bind to the plasma membrane in a palmitoylation-dependent manner (149). Also two effector proteins of the IpaH family of *Shigella* spp. were found to be *S*-palmitoylated in that study, suggesting that this modification can control the activity of effector proteins of other pathogens as well (149). Indeed, GobX and LpdA, effector proteins of *Legionella pneumophila*, the causative agent of Legionnaires’ disease invading macrophages and lung endothelial cells, are *S*-palmitoylated as was found recently using click chemistry. LpdA is a phospholipase hydrolyzing various phosphatidylinositols while GobX is an E3 ubiquitin ligase. GobX is targeted in a palmitoylation-dependent manner to the Golgi apparatus, and LpdA to the plasma membrane and a subset of intracellular vesicles (150, 151). Thus, the diversified subcellular localization of bacterial effector proteins reflects that of eukaryotic proteins.

It is worth noting that global profiling of acylated proteins with the application of click chemistry and an alkyne-functionalized analog of myristic acid, alk-14, for cell labeling was effective in reveling the mechanism of action of *Shigella flexneri* effector protein IpaJ of type III secretion system. This is a unique protease that cleaves off the N-terminal myristoylated glycine. This proteolytic demyristoylation activity of IpaJ is specific toward Golgi-associated ARF/ARL family of GTPases regulating cargo transport through the Golgi apparatus, inhibition of which is apparently pivotal for virulence of the bacteria causing diarrhea in humans (152).

In addition to the *S*-palmitoylation of the effectors of pathogenic bacteria of mammals mentioned earlier, double acylation, *N*-myristoylation and *S*-palmitoylation, has been reported of the so-called avirulence (Avr) proteins (effectors of type III secretion system) of *Pseudomonas syringae*, a causative agent of diverse plant diseases. Among them, AvrRpm1 and ArvB are *N*-myristoylated and *S*-palmitoylated by host acyltransferases at neighboring glycine and cysteine residues localized at the N-terminus of the proteins (similarly to eukaryotic kinases of the Src family), while in AvrPphB and two AvrPphB-like effectors—ORF4 and NopT, the double acylation motif is exposed after auto-cleavage of the proteins (similarly to some eukaryotic proteins cleaved by caspases). The acylation of the Avr proteins ensures their anchoring in the host plasma membrane, which is required for their functioning. In disease-susceptible plants Avr proteins contribute to successful infection; however, in plants expressing host resistance (R) genes they trigger plant defense signals, in both cases engaging plasma membrane-associated host proteins (153, 154).

The importance of palmitoylation of bacterial effector proteins for their infectivity is only beginning to be uncovered, in no small part owing to the development of the click chemistry-based method for detection of this protein modification. However, the strategy of high-jacking the host palmitoylation machinery to modify own proteins seems to be much more commonly employed by viruses.

## Protein Palmitoylation in Viral Infections

Viruses do not encode palmitoyl acyltransferases but exploit extensively the host palmitoylation machinery to modify their proteins essential for infection of host cells and own replication. In fact, *S*-palmitoylation of proteins was discovered in 1979 as a modification of envelope glycoproteins of Sindbis virus and VSV. In those studies [^3^H]-palmitic acid was used for metabolic labeling of virus-infected cells and labeled proteins were identified by autoradiography (12, 155). Subsequently, a number of other viral proteins have been found to be palmitoylated using this approach.

The most-studied group of viral palmitoylated proteins is those found in enveloped viruses, i.e., viruses covered by a lipid bilayer obtained during their replication from a membrane of the host cell, such as the plasma membrane or endoplasmic reticulum. Influenza virus, HIV-1, hepatitis C virus (HCV), and herpes simplex virus (HSV) are the best known enveloped viruses. The envelope is rich in transmembrane, often *S*-palmitoylated, glycoproteins called spikes, which can bind to cognate receptors on the host cell plasma membrane triggering endocytosis of the virion, mediate subsequent fusion of the viral and cellular membranes allowing entry of the viral genome to the cytoplasm, and are also involved in the budding of newly formed virus particles from the cell. An example of such multifunctional palmitoylated transmembrane glycoproteins is HA present in the envelope of influenza virus together with another palmitoylated transmembrane protein, the matrix protein M2, which forms a proton channel earning the protein the name viroporin. As mentioned earlier, HA of influenza A virus is *S*-stearoylated and *S*-palmitoylated, respectively, at one cysteine residue located in the transmembrane domain of HA and two cysteines found in the cytoplasmic (intraviral) tail in close proximity to the membrane (156). On the other hand, M2 is *S*-palmitoylated on the amphiphilic helix located in the cytoplasmic part of the protein. Due to the *S*-palmitoylation and the presence of a cholesterol-binding motif the helix bends toward and associates with membranes (157, 158). During infection, HA binds to sialic acid residues of glycans localized on the surface of airway and alveolar epithelial cells. The bound virions are endocytosed and next the viral and endosome membranes fuse. The membrane fusion is driven by HA, which undergoes conformational changes induced by low pH of endosomes. Acidification of endosomes activates also the M2 proton channel activity, protons entering viral core facilitate dissociation of the viral genome which then moves to the nucleus where RNA replication occurs. The *S*-palmitoylation of HA is required for the fusion of the viral and endosome membranes at least in some subtypes of the virus while the ion channel activity of M2 is not dependent on its *S*-palmitoylation (159). Newly synthesized viral proteins and RNA are assembled into virions in the plasma membrane rafts which merge into lager platforms crucial for the virion assembly and budding off. The triple fatty acylation of HA is required for its targeting to plasma membrane rafts (160, 161). Besides S-palmitoylation, also the amino acid sequence of the transmembrane domain of HA determines its association with rafts (45). On the other hand, among the amino acids of the cytoplasmic tail of HA no other than the two *S*-palmitoylated cysteines are required for viral assembly and replication, although it is still not clear whether raft targeting (in cooperation with the transmembrane fragment) is the only mechanism of their participation. It is proposed that they affect conformation of the HA tail controlling its interaction with structural matrix protein M1 lying beneath the viral envelope (162, 163). The budding off of the virion is facilitated by M2 which localizes at the edges of rafts as a result of a combination of its *S*-palmitoylation, cholesterol binding, and properties of the transmembrane fragment. M2 protein can create a “wedge” altering membrane curvature thereby facilitating membrane scission and release of the virion (157, 164).

The influenza virus *S*-palmitoylated proteins are the archetype for many other viral proteins. Thus, *S*-palmitoylated spike glycoproteins include S-protein of coronaviruses (e.g., severe acute respiratory syndrome virus), the fusion (F) protein of paramyxoviruses (e.g., measles virus), Env of retroviruses [e.g., HIV-1, feline immunodeficiency virus (FIV)], and filoviruses (e.g., Ebola). Other viral proteins modified with palmitate are viroporins, such as E protein of coronaviruses, and also peripheral membrane proteins or nucleocapsid proteins absent in influenza virus. It has been found that *S*-palmitoylation of F13L, a peripheral protein of the envelope of vaccinia virus, controls the association of the protein with intracellular membranes, thereby the formation of the envelope (165). The core protein of the nucleocapsid of HCV resides on the surface of lipid droplets and binds in a palmitoylation-dependent manner to membranes of the droplet-associated endoplasmic reticulum. Subsequently, it recruits viral proteins and newly synthesized RNA for viral particle formation (166). Besides the interest in the role of viral protein *S*-palmitoylation for infectivity and possible use of host zDHHC enzymes as targets of anti-influenza drugs (167), viral proteins often serve as a model to study the consequences of fatty acylation for protein functioning and localization in distinct membrane domains (see *S*-Palmitoylation of Proteins and Its Influence on Protein Localization, Trafficking, and Stability of this review). Readers are referred to recent exhaustive reviews that consider these topics (36, 84, 168) while we will focus here on the recent advances in the field of viral protein palmitoylation brought about mainly by proteomic studies.

The click chemistry-based approach has led to the identification of *S*-palmitoylation in the cytoplasmic domain of the transmembrane spike protein Env of FIV, considered to be the cat equivalent of HIV-1. Env comprises three transmembrane gp41 glycoproteins and three associated gp120 which bind to CD4 receptor and coreceptors on the surface of T lymphocytes allowing fusion of the viral envelope and the plasma membrane and entry of viral capsid. Four cysteines in FIV Env are *S*-palmitoylated *vis-a-vis* two found in the Env of HIV-1. The two most membrane-proximal cysteines, 804 and 811, are required for the FIV membrane-fusion activity and incorporation of Env into virions (169), in agreement with the importance of Env *S*-palmitoylation for virion assembly of some HIV-1 strains (170–172). The assembly of HIV-1 virions takes place in plasma membrane rafts and is driven by *N*-myristoylated Gag protein which anchors and oligomerizes preferentially in these plasma membrane domains due to the presence of the fatty acyl chain (18).

The development of click chemistry-based methods allowed for the first time global profiling of acylated proteins in virus-infected cells. In addition to identifying acylated viral proteins this approach has also revealed how the viral infection modulates the acylation pattern of the host cell proteins. Thus far, click chemistry has been used to study protein myristoylation and palmitoylation in cells infected with HIV-1 and with HSV. In the latter case, the standard metabolic labeling with alkyne-functionalized myristic and palmitic acid analogs followed by click chemistry and mass spectrometry was combined with SILAC to discern between the changes in the extent of protein acylation and those in their abundance following viral infection. This approach allowed an elaborate quantitative analysis of host protein acylation and has revealed an overall downregulation of the level of both host protein modifications in infected cells. While the decreased content of myristoylated proteins resulted mainly from suppression of host protein synthesis, the drop in several *S*-palmitoylated proteins ensued from the inhibition of their palmitoylation in infected cells. The affected proteins were localized mainly to the plasma membrane and the Golgi apparatus and were involved in vesicle-mediated transport and ion transport. In addition, the study has expanded the list of HSV-encoded acylated (mostly palmitoylated) proteins that play different functions in the viral cycle, such as gE, gI, gK, US2, and US3 (110). Similar results pointing to global changes of host protein acylation were obtained upon analysis of protein myristoylation and palmitoylation in cells infected with HIV-1. In that study, the cells were labeled with analogs of palmitic or myristic acid tagged with an azide moiety for click chemistry reaction; however, the following mass spectrometry analysis did not address the relation between changes of protein acylation vs. alteration of protein level. The study identified 17 palmitoylated and 7 myristoylated proteins significantly differing in abundance between HIV-1 infected and uninfected cells. Several of the proteins affected by the infection were of host origin. The abundance of myristoylated proteins was in general increased while that of the palmitoylated ones—decreased in infected cells (173). In other words, the two studies have revealed that HSV and HIV-1 not only encode proteins that are acylated in the host cell but also alter the palmitoylation of host proteins, likely to adapt the cellular environment to favor their replication and budding. The majority of the acylated proteins affected by HIV-1 or HSV infection had not been described earlier in this context; therefore, further studies on these proteins could be crucial for better understanding of viral infection. Thus, the click chemistry-based approach has been highly effective in revealing changes of the host protein palmitoylation and opening new possibilities for the identification of novel antiviral drug targets.

## Palmitoylation of Host Proteins Involved in Antibacterial and Antiviral Defense

The innate immune responses are the first line of active defense against microbial infections. The application of click chemistry-based and ABE methods and their use for large-scale analysis of protein palmitoylation in murine dendritic CD2.4 cells (10, 103), and murine macrophage-like RAW264 cells (14, 48) complemented by proteomic analysis of the raft fraction of those cells (47) have contributed significantly to the understanding of the role of palmitoylation of host receptors and signaling proteins involved in innate immune responses. Thus, the palmitoyl proteome analysis of murine dendritic cells unraveled *S*-palmitoylation of TLR2, a receptor expressed in cells of myeloidal lineage, which heterodimerizes with TLR1 or TLR6 to bind bacterial tri- or diacylated lipoproteins, respectively, and also other microbial components, such as glycolipids (e.g., lipoarabinomannan) of *Mycobacterium* and yeast zymosan (174). Besides TLR2, two other human TLRs out of 10 ectopically expressed in HEK293 cell, flagellin receptor TLR5, and TLR10, a unique TLR negatively regulating the pro-inflammatory activity of TLR2, were also found to be palmitoylated. The *S*-palmitoylation site of human TLR2 was mapped to Cys609 adjacent to its transmembrane domain. The modification was present in unstimulated cells and was linked with up-regulation of the cell surface localization of TLR2. Mutation of Cys609 abolished the ability of the receptor to induce pro-inflammatory signaling in response to microbial ligands of TLR2 (10). Further studies are needed to reveal whether *S*-palmitoylation of TLR2 controls its association with rafts as sites of TLR2 activation (175) and/or affects endocytosis of the receptor, as found for the anthrax toxin receptor (54).

One of the most extensively studied TLRs, TLR4 activated by bacterial LPS, is not palmitoylated. Yet, saturated fatty acids have been indicated to trigger pro-inflammatory signaling of TLR4. Thus, the TLR4/MD2 receptor complex is involved in the pro-inflammatory outcome of a diet rich in palmitic acid, as was found when analyzing markers of inflammation in the heart and adipose tissue of high fat diet-fed mice (176, 177). The molecular mechanisms underlying the pro-inflammatory properties of palmitic acid can involve its influence on the plasma membrane lipid order, hence raft organization, in a way that facilitates translocation of TLR4 (and TLR2) toward rafts (178, 179). Palmitic acid also directly binds to the TLR4-associated MD2 protein (177, 180). An influence of palmitic acid on sphingomyelin/ceramide metabolism, which enhances the LPS-induced responses, has also been considered (181). Recent proteomic studies based on 17ODYA labeling of RAW264 macrophage-like cells followed by click chemistry have revealed that stimulation of cells with LPS induces profound changes of the abundance of palmitoylated proteins (182). The data are in agreement with earlier findings showing that LPS induces accumulation of *S*-palmitoylated Lyn kinase in the raft-enriched fraction of cells, allowing it to downregulate TLR4 signaling (11). One of the upregulated *S*-palmitoylated proteins was type II phosphatidylinositol 4-kinase IIβ, which phosphorylates phosphatidylinositol to phosphatidylinositol 4-monophosphate. It was shown that palmitoylation determines the involvement of the kinase in LPS-induced signaling (182). These data suggest that *S*-palmitoylated proteins, including enzymes catalyzing phosphatidylinositol synthesis and turnover, are important factors affecting the pro-inflammatory responses triggered by LPS.

**Figure 3:**
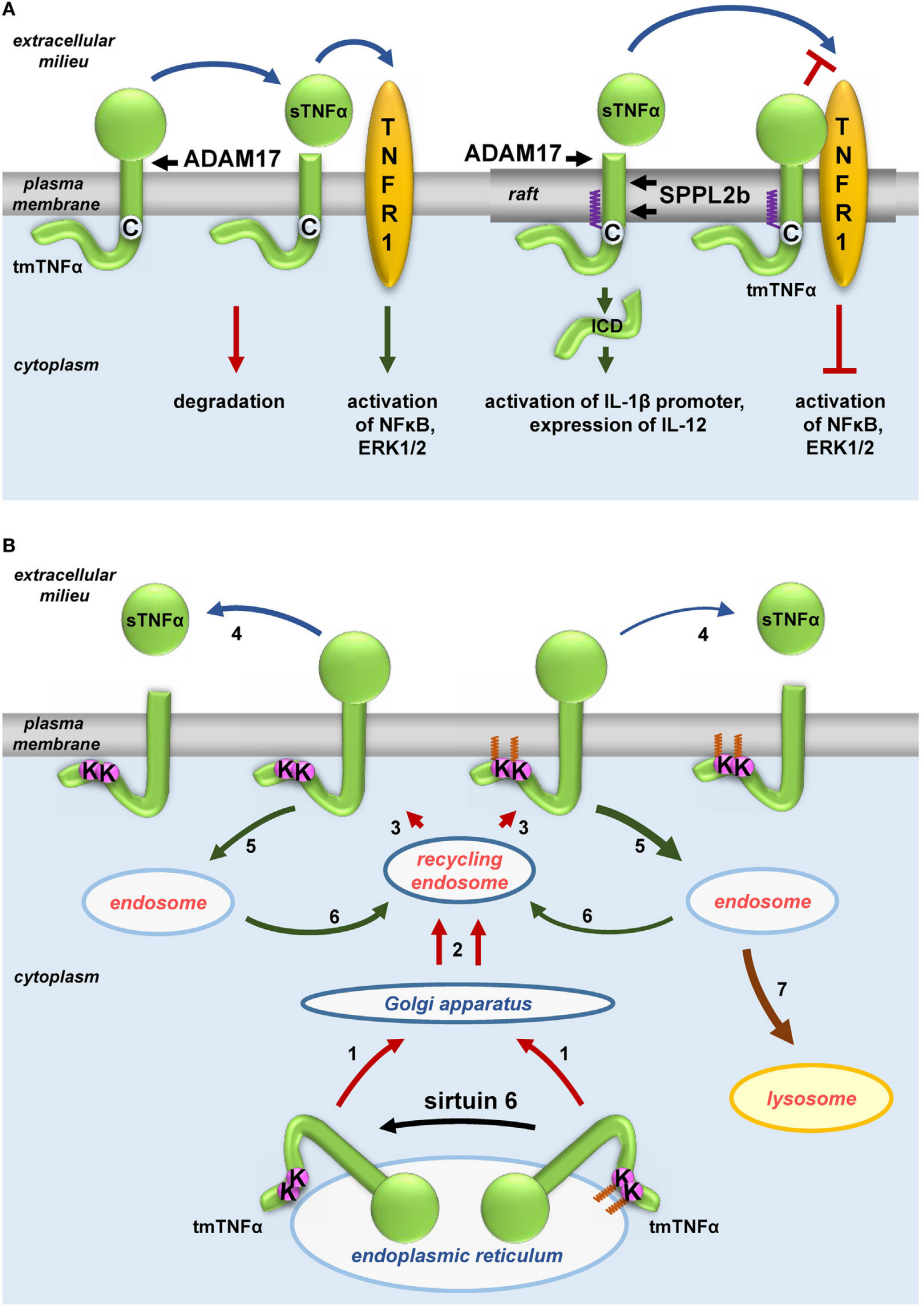
Influence of fatty acylation of transmembrane tumor necrosis factor α (TNFα) on production of soluble sTNFα. **(A)** *S*-palmitoylation and **(B)** ε-*N*-myristoylation of tmTNFα. **(A)** Non-palmitoylated tmTNFα is localized outside rafts while that *S*-palmitoylated on Cys30—in rafts of the plasma membrane. tmTNFα is cleaved by ADAM17 protease in both these plasma membrane environments giving rise to sTNFα, which subsequently activates TNF receptor (TNFR) 1 receptor leading to activation of NFκB and ERK1/2. However, only the raft-residing tmTNFα is further processed by SPPL2b protease to yield ICD, which activates the promoter of interleukin (IL)-1β and expression of IL-12. On the other hand, a pool of *S*-palmitoylated tmTNFα interacts in rafts with TNFR1 preventing its activation by sTNFα. **(B)** tmTNFα is transported from the endoplasmic reticulum *via* Golgi apparatus and recycling endosomes [1, 2] to the plasma membrane [3]. In the plasma membrane, TNFα is cleaved by ADAM17 giving rise to sTNFα [4] or is internalized [5] and either returns from the endosomes to the plasma membrane [6, 3] or is directed to lysosomes for degradation [7]. ε-*N*-myristoylation of tmTNFα at Lys19 and Lys20 facilitates its degradation [5, 7] at the expense of processing to sTNFα [4]. Oligomerization of tmTNFα and TNFR1 is not shown.

Notably LPS induces production of TNFα, a pro-inflammatory cytokine that is *S*-palmitoylated itself. TNFα is synthesized as a transmembrane 27-kDa precursor (tmTNFα) transported from the endoplasmic reticulum to the plasma membrane through the Golgi apparatus and recycling endosomes (183). Human tmTNFα is *S*-palmitoylated at Cys30 located at the boundary between its transmembrane and cytosolic fragments, as was found independently by radiolabeling and by labeling with 17ODYA followed by click chemistry (184, 185). Poggi et al. (185) arrived at a complex model explaining how the *S*-palmitoylation of TNFα affects its activity (Figure 3A). The modification was shown to favor the association of tmTNFα with rafts. Upon cell activation, the extracellular domain of tmTNF is cleaved by ADAM17 metalloproteinase whereupon the soluble TNFα (sTNFα) is released to the extracellular milieu and activates TNF receptor (TNFR) 1 and TNFR2. As ADAM17 localizes to both non-raft and raft regions of the plasma membrane, the *S*-palmitoylation of tmTNFα does not affect its cleavage and production of the soluble cytokine. However, *S*-palmitoylated tmTNFα interacts with TNFR1 in rafts thereby reducing the binding of sTNFα and consequently reducing the sensitivity of the cell to this cytokine. In addition, the fragment of tmTNFα which remains after the release of sTNFα in rafts if further processed by intramembrane SPPL2a and 2b proteases giving rise to ICD (intracellular domain) of an own biological activity. By contrast, the non-raft fragment of the ADAM17-cleaved tmTNFα is rapidly degraded (185).

The transport and maturation of TNFα are also regulated by another posttranslational acylation, ε-*N*-myristoylation (22). As shown in Figure 3B, myristic acid residues are attached to two lysines (Lys19 and 20) of human tmTNFα. This modification is reversed by sirtuin 6 catalyzing the demyristoylation. Depletion of sirtuin 6 decreases the release of sTNFα since the ε-*N*-acylated TNFα precursor is redirected to and accumulates in lysosomes (90, 91). It is worth noting that exogenous palmitic acid stimulates the ε-*N*-myristoylation of tmTNFα, thereby reducing the release of sTNFα in favor of accumulation of tmTNFα in lysosomes (90, 91). This somehow surprising anti-inflammatory effect of palmitic acid can be explained by competitive binding between long-chain fatty acids (in this case, palmitic) and myristoylated substrates of sirtuin 6 found *in vitro*—(89) and adds a new dimension to the potential effects of palmitic acid.

*S*-palmitoylation of host proteins is also vital in antiviral defense. Viral nucleic acids, which are recognized by several TLRs and also cytoplasmic pattern-recognition receptors, induce robust production of type I interferons (IFNs), mainly INFα and IFNβ. The IFNα and IFNβ released from cells which first encounter viruses, e.g., dendritic cells, induce an antiviral reaction in an autocrine and paracrine manner upon binding to plasma membrane IFNα/β receptor (IFNAR) consisting of subunits 1 and 2. Both human IFNAR subunits are *S*-palmitoylated, as has been found by classical radiolabeling. The *S*-palmitoylation of IFNAR1 on Cys463, localized near the cytoplasmic end of the transmembrane domain, is required for downstream activation of STAT1 and STAT2 and the following transcription of IFNα-activated genes (186). Among the IFN-induced proteins, some have been shown to be palmitoylated, using click chemistry and ABE. They include the immunity-related GTPase Irgm1, BST2 also known as tetherin, and IFITM1 and 3 (10, 104). IFITMs are potent restriction factors against a wide range of enveloped viruses, e.g., influenza, West Nile, dengue, and Zika viruses (187, 188). IFITMs localize primarily to endolysosomal membranes where they inhibit viral replication by blocking their fusion with these membranes and also facilitate virus degradation (187). The exact mechanism of this antiviral activity is not clear, but it seems to rely on a perturbation of the organization of endolysosomal membranes. This can be linked with the intramembrane topology of IFITMs and their *S*-palmitoylation. IFITM1 and 3 likely possess two loops embedded in but not spanning the membrane with both the N- and C-termini facing the cytoplasm (55, 189). *S*-palmitoylation of conserved cysteine residues adjacent to these loops, Cys71, 72, and 105 in murine IFITM3, contributes to the membrane binding, similarly as found earlier for caveolins (119, 189). The *S*-palmitoylation also facilitates clustering of IFITM3 in the membranes, which is of potential significance for its antiviral activity (103). In support of the latter, the antiviral capacity was markedly reduced for non-palmitoylated mutant forms of IFITM3 (103, 119). However, *S*-palmitoylation did not affect the endolysosomal localization or stability of IFITM3. Subsequent studies have revealed that the localization and degradation of murine IFITM3, both shaping its antiviral capacity, are orchestrated by numerous posttranslational modifications comprising polyubiquitination, tyrosine phosphorylation by the Src-family kinase Fyn, and methylation (189, 190). By contrast, *S*-palmitoylation alone of the closely related murine IFITM1 endowed it with an antiviral activity and enhanced stability by preventing proteasomal degradation (55), which indicates diverse effects of this modification on individual IFITM isoforms.

The presented data are only beginning to fill the gap which existed in our understanding of the role of protein palmitoylation in innate immune responses. For a long time, it was lagging behind that on acquired immune responses, in which a plethora of *S*-palmitoylated proteins have long been known to be involved. They include receptors (CD4 and CD8), tyrosine kinases of the Src family, transmembrane adaptor proteins (e.g., LAT, NTAL, and PAG/Cbp), and α subunits of heterotrimeric G proteins. Their *S*-palmitoylation in most cases targets them to rafts and is a prerequisite for their involvement in the signaling pathways triggered by immunoreceptors [TCR, B cell receptor (BCR), and Fcγ and Fcε receptors] crucial for the acquired immune responses. An association of some components of these signaling pathways with tetraspanin-enriched domains has also been considered. These topics are discussed in several earlier reviews (44, 79, 191, 192). It is worth noting that large-scale proteomic analyses of fatty-acylated proteins of T cells (99, 104, 105, 122) and B cells (121), identifying numerous new palmitoylated proteins, have been published recently. Further studies will shed light on the possible engagement of those proteins in acquired immune responses and/or in the cross talk between the innate and the acquired immune system, in which phagocytic cells, such as macrophages and dendritic cells, are essential (193).

## Concluding Remarks

Protein *S*-palmitoylation affects their localization, trafficking, and stability. It has long been known as an important factor controlling signal transduction by the BCR and TCR receptors involved in acquired immune responses. It is now becoming evident that palmitic acid is also a key lipid affecting the diverse processes at the host–pathogen encounter. Palmitate is a component of bacterial LPS and lipoproteins; *S*-palmitoylation of viral, some bacterial, and numerous host proteins is recognized as a crucial factor affecting both the virulence of pathogens and the innate immune reactions of the host. Our understanding of the latter has benefited greatly from the development of novel methods of detection of this protein modification. Their application has led to the identification of numerous proteins involved in the host–pathogen interaction. The methods have also allowed high-throughput proteomic analysis of palmitoylation of proteins in infected cells, showing widespread changes of the host cell palmitoylome. Future studies will tell whether complex feedback loops comprising palmitoyl acyltransferases and acylthioesterases, similar to those of kinases and phosphatases carrying out protein phosphorylation/dephosphorylation, are involved in controlling protein *S*-palmitoylation in infected cells. Revealing how the *S*-palmitoylation of particular proteins is regulated during the host–pathogen interactions should allow its modulation to favor the host defense.

## Author Contributions

All authors contributed to writing and critically revised the paper.

## Conflict of Interest Statement

The authors declare that the research was conducted in the absence of any commercial or financial relationships that could be construed as a potential conflict of interest.

## Abbreviations

17ODYA, 17-octadecynoic acid; ABE, acyl-biotin exchange; APT, acyl-protein thioesterase; BCR, B cell receptor; FIV, feline immunodeficiency virus; GPI, glycosylphosphatidylinositol; HA, hemagglutinin; HCV, hepatitis C virus; HIV-1, human immunodeficiency virus-1; HSV, herpes simplex virus; IFITM, interferon-induced transmembrane protein; IFN, interferon; IFNAR, IFNα/β receptor; IL, interleukin; LPS, lipopolysaccharide; PPT, palmitoyl protein thioesterase; SILAC, stable isotope labeling by amino acids in cells; SILAM, stable isotope labeling of mammals; TCR, T cell receptor; TEM8, tumor endothelial marker 8; TNF, tumor necrosis factor; TNFR, TNF receptor; TLR, toll-like receptor; VSV, vesicular stomatitis virus; zDHHC, zinc finger DHHC domain containing.
